# Supplementary material for: DMRforPairs: identifying Differentially Methylated Regions between unique samples using array based methylation profiles
Source: BMC Bioinformatics. 2014 May 15;15:141. doi: 10.1186/1471-2105-15-141 (PMC4046028; doi:10.1186/1471-2105-15-141)
Supplement: Additional file 2 — DMRforPairs output for the comparison of A431-MCF7 and NA17018-NA17105. Please start from the HTML files in each folder. Available via the BMC Bioinformatics website. [file 1471-2105-15-141-S2.zip › 1394847754114233_MOESM2_ESM/A431_MCF7/figures/6490.pdf]

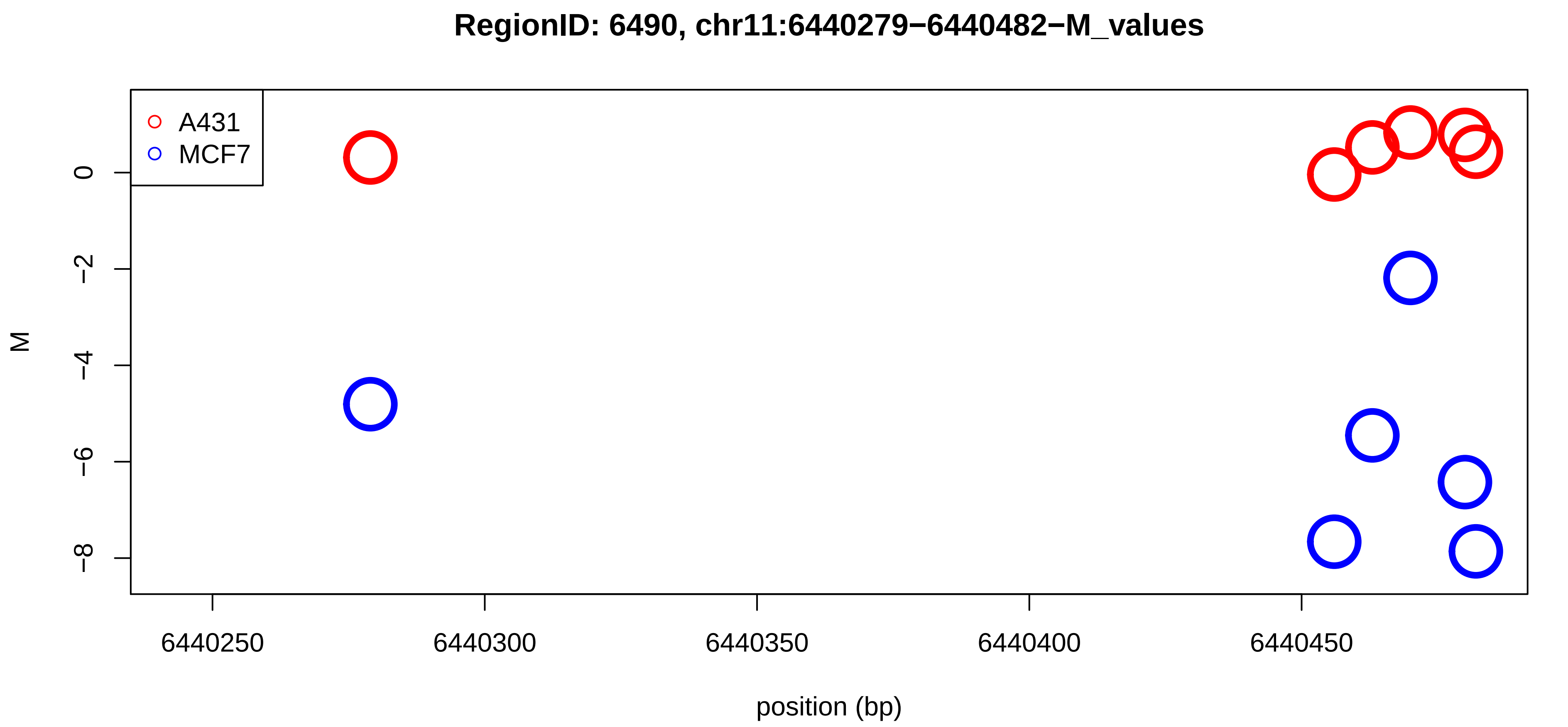

RegionID: 6490, chr11:6440279−6440482−M_values
A431
MCF7
M
0
−2
−4
−6
−8
6440250
6440300
6440350
6440400
6440450
position (bp)

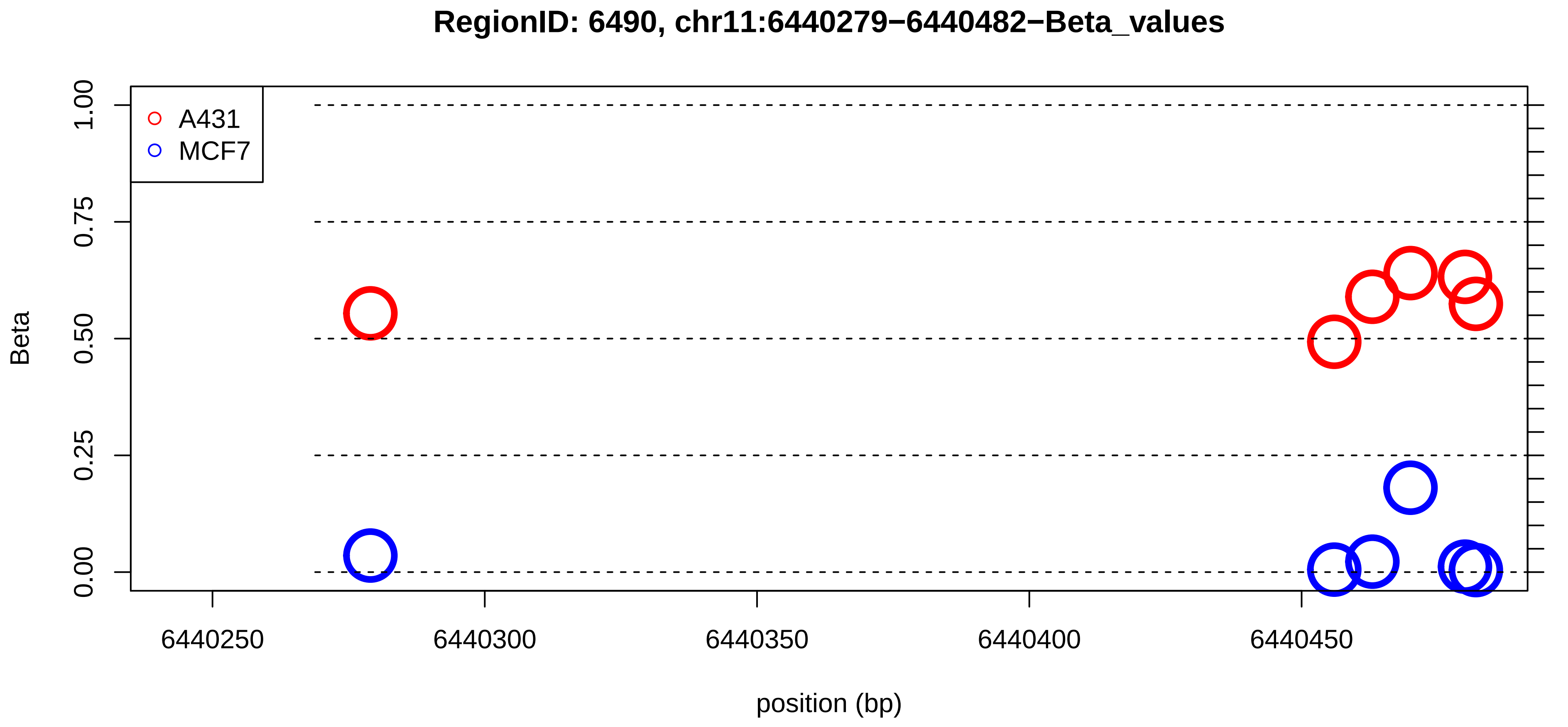

RegionID: 6490, chr11:6440279−6440482−Beta_values
A431
MCF7
Beta
1.00
0.75
0.50
0.25
0.00
6440250
6440300
6440350
6440400
6440450
position (bp)

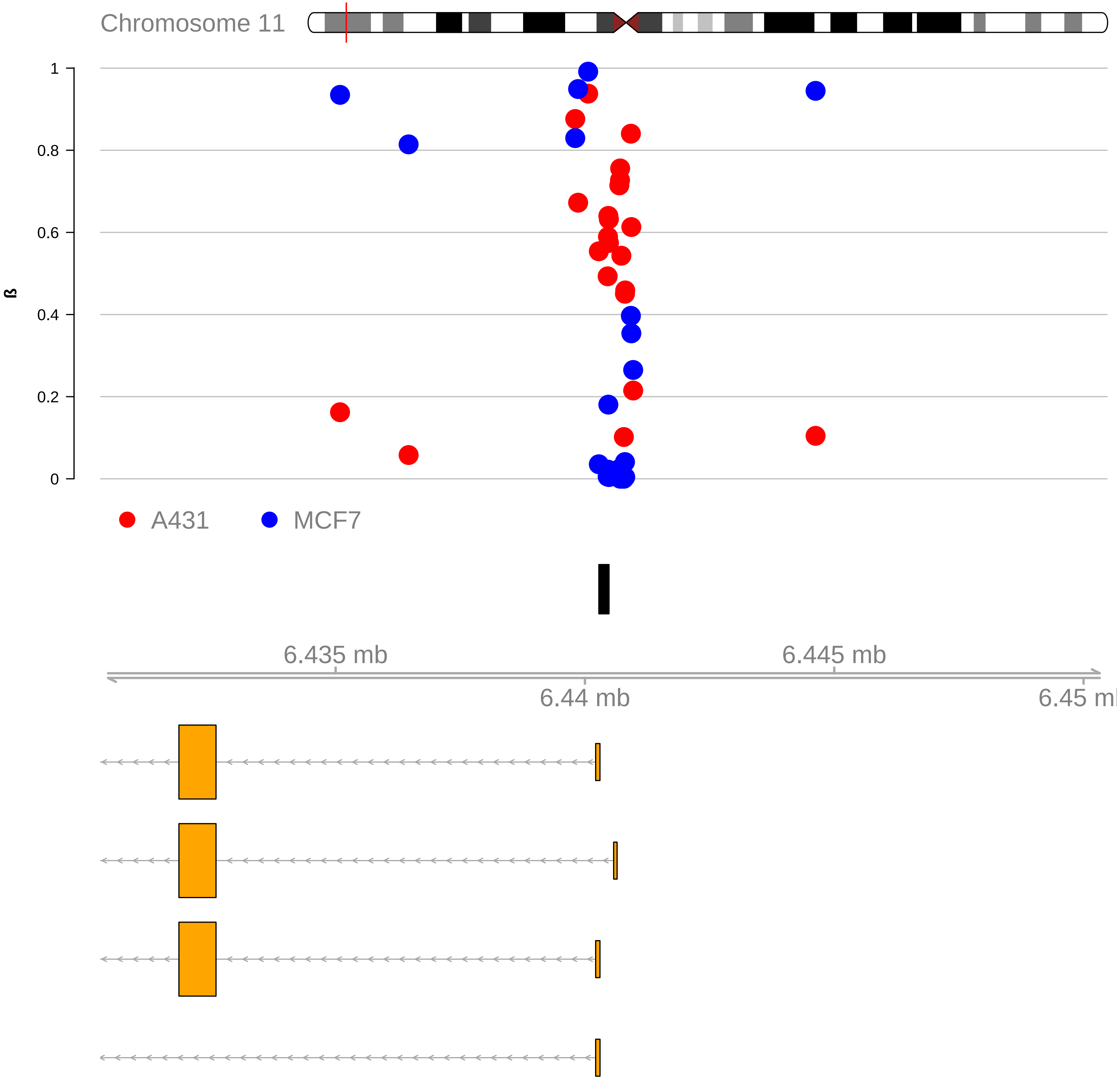

Chromosome 11
1
0.8
0.6
0.4
0.2
0
ß
A431
MCF7
6.435 mb
6.445 mb
6.44 mb
6.45 mb
